# Study on Antiphospholipid/Anticardioliplin Antibodies in Women With Recurrent Abortion

**DOI:** 10.5812/ircmj.4857

**Published:** 2013-08-05

**Authors:** Farideh Akhlaghi, Mohamad Reza Keramati, Mehri Tafazoli

**Affiliations:** 1 Faculty of Medicine, Mashhad University of Medical Sciences, Mashhad, IR Iran

**Keywords:** Antiphospholipids, antibodies, Anticardiolipin Antibodies, Recurrent Abortion

## Abstract

**Background:**

Antiphospholipid antibodies are associated with recurrent abortion but correlation between level of antibodies and gestational age of abortion and duration post abortion is not clear.

**Objectives:**

Aim of this study was study on relation between antiphospholipid antibodies in women with recurrent abortion and their gestational age and duration post abortion.

**Patients and Methods:**

We performed a case-control study on 197 pregnant women who had history of spontaneous recurrent abortion as case group and 50 pregnant healthy women as control group. Demographic characteristic of all participants filled in questionnaire forms. Antipospholipid and anticardiolipin antibodies were measured in their serum by Enzyme linked Immunoassay with orgenec kits. Data analyzed by SPSS software (version 13) and T statistical test. P value less than 0.05 was considered significant.

**Results:**

Mean age of participants was 24-39 years old. The average rate of antiphospolipid antibodies in patients with normal anticardiolipin was greater than those with abnormal anticardiolipin and T-test showed significant difference between two groups.(P = 0.000) In case group the number of abortions was more, mean of antiphosopolipid antibody levels were also higher. Mean anticardiolipin and antiphospholipid antibodies rate was greater with increasing gestational age at time of first abortion. Almost mean antipospholipid and anticardiolipin antibodies in all patients remained in high level just in first 5 years with any number of abortions and five years later, antibodies began to fall.

**Conclusions:**

Antipospholipid antibodies based on number of abortions and gestational age of abortions were increased. Mean antipospholipid and anticardiolipin antibodies in all patients remained in high level just in first 5 years post abortion and then began to fall.

## 1. Background

Recurrent pregnancy loss is secondary to multiple illnesses. An important cause sometimes undiagnosed is the antiphospholipid syndrome, an autoimmune disease with various clinical alterations (1). Antiphospholipid syndrome has received considerable attention from the medical community because of its association with a number of serious clinical disorders (2). Antiphospholipid antibody syndrome is characterized by autoantibodies against negatively charged phospholipids in the serum, and clinically by multiple thromboses, thrombocytopenia, and recurrent fetal loss. The mechanism by which the antibodies cause the clinical picture are not clear (3).

The antiphospholipid syndrome was reported in the early 1980s as the association of thrombosis, recurrent pregnancy loss in the presence of anticardiolipin antibodies and/or lupus anticoagulant. Since then, many other clinical manifestations have been associated with antiphospholipid. Almost any organ and tissue may be involved in the disease, including the brain, the heart, the kidneys, the placenta and many more (4). Antiphospholipid syndrome is characterized by recurrent arterial or venous thromboembolism or pregnancy loss in association with antibodies directed against anionic phospholipids or plasma proteins bound to anionic phospholipids. In accordance with this, fetal abortion, typically beyond the tenth week of gestation, is also caused by infarctions of blood vessels in the placenta (5). Among the autoimmune factors, anti-phospholipid antibodies have been demonstrated to be the strongest risk factors for foetal loss, the prevalence of which is as high as 40% in women with recurrent fetal loss (6). But the pathophysiologic mechanisms that characterize thrombosis and recurrent pregnancy losses are still not clear (7). Thrombotic events at the placental level cannot explain all of the clinical manifestations. It has been suggested that anticardiolipin may be responsible for a local acute inflammatory response mediated by complement activation and neutrophil infiltration eventually leading to fetal loss (8). Obstetric complications such as fetal death, premature delivery, preeclampsia and recurrent abortions are characteristic manifestations of antiphospholipid syndrome (9). The antiphospholipid antibody syndrome is an autoimmune condition in which vascular thrombosis and/or recurrent pregnancy losses occur in patients (10) and are risk factors for recurrent pregnancy loss and obstetric complications (11) which is characterized by recurrent fetal loss, thrombosis, and thrombocytopenia in association with anticardiolipin antibodies (12).

## 2. Objectives

The aim of the study was to evaluate the prevalence of antiphospholipid/anticardiolipin antibodies among women with recurrent abortion and to determine any association between antiphospholipid/anticardiolipin antibodies and number of abortion and duration post abortions. 

## 3. Patients and Methods

In this case control study 247 pregnant women with and without history of recurrent abortions participated. Women who referred to prenatal care clinic of Zeinab hospital who had inclusion criteria and satisfied and signed the consent form were enrolled in this study. Inclusion criteria included history of two or more abortions, no history of medical diseases and drug use. Abortion means fetal loss before 20 weeks of gestational age or fetal weight lower than 500 gram. 197 women with history of abortions who had inclusion criteria selected as case group and 50 healthy women without history of abortions and had one or more successful pregnancies selected as control group. First demographic characteristic of participants filled in questionnaires forms and obstetrics history include parity, live birth, number of abortions, gestational age at each abortion and duration post abortions were determined. Then blood samplings were done for all participiants in both groups and serums of samples were separated and were kept at -20 °C temperature. At the end of each week collected samples were sent to Emam Reza laboratory and level of antiphospholipid and anticardiolipin antibodies in samples were measured by enzyme linked immunoassay test and use of Orgentec kits. Data were analyzed by using SPSS software (version 13) and statistical tests in case and control groups and relation between mean of antiphospolipid/anticardiolipin antibodies and number of abortions (2 or ≥ 3) , gestational age at the time of abortions (≤ 8 week, 9 – 16 weeks, > 16 week) and duration post abortions (≤ 5 year, 6 – l0 years, > 10 years) were determined. P value less than 0.05 was considered significant.

## 4. Results

Mean age of all participants was 24 – 39 years old. The results of this study showed the average rate of antiphospolipid antibodies in patients with normal anticardiolipin was greater than those with abnormal anticardiolipin and T-test was showed significant difference between two groups (Table 1). Mean anticardiolipin rate in case group was lower than control group, but antiphospholipid rate in case group was equal with control group and by using T-test two groups had not significant statistically differences (Table 2). Mean antiphospholipid in patients based on previous history of abortions showed that whatever the number of previous abortions was more, antiphospolipid rate was higher, but anticardiolipin rate in whom did not increase (Table 3). Mean anticardiolipin and antiphospholipids rates based on gestational age in patients indicated that with increasing gestational age at the time of first abortion, mean level of two antibodies were higher. Based on gestational age of second abortion, mean of antiphospholipid distribution was almost equal in all groups but the distribution of mean anticardiolipin in women who had abortions less than eight weeks was higher than other groups. Mean anticardiolipin distribution not related to the gestational age of third abortion, but the average distribution of antiphospholipid by gestational age showed that highest rate was seen in abortions between 9-16 weeks of pregnancy (Table 4). 

**Table 1.** Distribution of Mean Antipospholipid Antibodies in Patients with Normal and Abnormal Anticardiolipin Antibodies

Anticardiolipin Antibodies	Number	Mean Antipospholipid Antibodies	Standard Diviation
**Abnormal**	10	23.5270	21.25578
**Normal**	182	4.3607	3.21942
**P value**	T = 10.552, P = 0.000		

**Table 2.** Distribution of Mean Antipospholipid Antibodies and Anticardiolipin Antibodies in Case and Control Groups

Group	Number	Anticardiolipin Antibodies	Standard Deviation	Mean Antipospholipid	Standard Deviation
**Case**	154	06/2	4.99	5.41	6.3
**Control**	38	3.09	6.12	5.11	9.4
**P value**		T = 0.18, P = 0.85		T = 0.23, P = 0.81	

**Table 3.** Average Distribution of Antiphospholipid and Anticardiolipin Antibodies based on the Number of Previous Abortions

History of Previous Abortion	Antiphospholipids, No. (Mean ± SD)	Anticardiolipin, No. (Mean ± SD)
**Two**	73 (4.61 ± 3.3)	73 (1.59 ± 3.25)
**Three and more**	50(5.71 ± 9.4)	50 (1.65 ± 6.6)
**P Value**	T = 0.35, P = 9.1	T = 0.95, P = 0.06

**Table 4.** Average Distribution of Antiphospholipid and Anticardiolipin Antibodies based on Gestational Age of Different Abortions

Gestational age of Abortion	Antiphospholipids			Anticardiolipin		
	First Abortion, No. (Mean ± SD)	Second Abortion, No. (Mean ± SD)	Third Abortion, No. (Mean ± SD)	First Abortion, No. (Mean ± SD)	Second Abortion, No. (Mean ± SD)	Third Abortion, No. (Mean ± SD)
**≤ 8 week**	63 (4.48 ± 3.2)	65 (5.66 ± 8.67)	20 (5.7 ± 4.2)	63 (1.32 ± 3.16)	65 (2.33 ± 6.80)	20 (1.75 ± 5.3)
**9-16 Weeks**	55 (5.85 ± 9)	45 (5.01 ± 3.07)	27 (6.5 ± 12.6)	55 (2.14 ± 6.45)	45 (1.84 ± 2.60)	27 (2.58 ± 8.96)
**> 16 Weeks**	10 (7.13 ± 4.9)	10(4.69 ± 2.33)	3 (4.1 ± 2.8)	10 (4.37 ± 7.7)	10 (0.94 ± 1.84)	3 (0.71 ± 1.14)
**P Value**	F = 0.34, P = 0.08	F = 0.84, P = 0.17	F = 0.09, P = 0.90	F = 0.21, P = 1.5	F = 0.57, P = 0.55	F = 0.87, P = 0.13

Mean of antiphospholipid distribution in patients with less than five years after their first abortion was slightly more than those with more than 5 year after their abortion. Mean antiphospholipid rates in women with less than five years past their second abortion almost was equal to those of more than five years but mean anticardiolipin in whom with 6 – 10 years past after their second abortion was much higher than other groups. Distribution of mean anticardiolipin in women with less than five years past their third abortion was much higher than those with more than five years but mean of antiphospholipid distribution in women whom with less than five years past of their third abortion was equal to those with more than five years (Table 5). 

**Table 5.** Average Distribution of Antiphospholipid and Anticardiolipin Antibodies based on Duration Past of Different Abortions

Duration Post Abortion	Antiphospholipids			Anticardiolipin		
	First Abortion, No. (Mean ± SD)	Second Abortion, No. (Mean ± SD)	Third Abortion, No. (Mean ± SD)	First Abortion, No. (Mean ± SD)	Second Abortion, No. (Mean ± SD)	Third Abortion, No. (Mean ± SD)
**≤ 5 Years**	88 (5.49 ± 7.4)	108 (5.15 ± 6.83)	41 (6.57 ± 10.53)	88 (2.25 ± 5.77)	108 (1.93 ± 5.28)	41 (2.50 ± 7.99)
**6 – 10 Years**	26 (4.5 ± 4.1)	9 (5.81 ± 5.99)	6 (3.13 ± 1.61)	26 (1.38 ± 4.49)	9 (2.65 ± 7.51)	6 (0.19 ± 0.21)
**> 10 Years**	14 (4.8 ± 3)	6 (5.38 ± 2.70)	2 (3.89 ± 3.74)	14 (0.14 ± 0.10)	6 (0.18 ± 0.13)	2 (0.28 ± 0.22)
**P Value**	F = 0.78, P = 0.24	F = 0.95, P = 0.04	F = 0.69, P = 0.36	F = 0.33, P = 1.11	F = 0.67, P = 0.4	F = 0.73, P = 0.31

## 5. Discussions

Antiphospholipid syndrome is characterized by recurrent arterial or venous thromboembolism or pregnancy loss in association with antibodies directed against anionic phospholipids or plasma proteins bound to anionic phospholipids. A common cause of the huge variety of clinical manifestations is vaso-occlusive disease and not vasculitis in venous or arterial blood vessels of different sizes. In accordance with this, fetal abortion, typically beyond the tenth week of gestation, is also caused by infarctions of blood vessels in the placenta (5). Despite the strong association between antiphospholipid and thrombosis, the pathogenic role of antiphospholipid in the development of thrombosis has not been fully elucidated (4). Giasuddin (13) resulted the prevalence of anticardiolipin antibodies varies according to population being 37.1% in his patients with recurrent pregnancy loss and 5.4% in controls. Velayuthaprabhu et al. (12) in their study evaluated the anticardiolipin antibodies and antiphosphatidyl serine antibodies in women with recurrent abortion and they concluded that anticardiolipin antibody is found to be the most important factor for recurrent abortion. But we found mean anticardiolipin rate in patient group was less than control group, but antiphospholipid rate in case and control groups was equal. On the other hand results of study that was done by Tebo et al. (14) was agreeing with our results. They concluded that overall combined sensitivity of non-recommended antiphospholipid assays was not significantly higher than anticardiolipin tests and multiple antiphospholipid specificities in recurrent pregnancy loss group is not significantly different from control group and therefore no clinical significance. So Salmon (15) studied about the role of complement in the antiphospholipid syndrome and founded that complement activation is a central mechanism in antiphospholipid antibody-induced pregnancy loss and fetal growth restriction. In contrast of our results which anticardiolipin antibodies was lower in case group than control group, Sheth (16) observed in his study that anticardiolipin is a major cause of recurrent fetal loss and many pregnancies can be saved if diagnosed and treated adequately. Also Ghosh (17) in a prospective, observational study evaluated the prevalence of antiphospholipid syndrome among women with recurrent miscarriages/late pregnancy loss and 27.7% were positive for antiphospholipid antibodies but we found antiphospholipid rate in case and control groups was equal. Bagger et al. (18) investigated to which degree of IgG, IgA and IgM anticardiolipin antibodies are associated with recurrent abortion and Dr Shetty also studied about antiphospholipid antibodies and other immunological causes of recurrent fetal loss. He found Low-molecular-weight heparin with low-dose aspirin is the most effective treatment for women with antiphospholipid antibodies syndrome and recurrent fetal loss. Differences in dosage, timing of treatment, inclusion criteria and outcome assessment parameters are some of the factors which have resulted in discrepancies in various reports. He recommended identification of immunological mechanisms involved in pregnancy loss and the action of different therapeutic reagents is important so that effective therapies can be designed and investigated (6). Dr Bates (19) believed that the antiphospholipid antibodies seem to be clearly associated with recurrent miscarriage. Although there is no agreement on the mechanisms of recurrent pregnancy loss in patients with these antibodies, vasculopathy of terminal spiral arteries may be implicated and there is a general consensus to routinely screen for antiphospholipid antibodies in patients with recurrent miscarriage. Well-designed diagnostic studies are needed to estimate the true association between other specific autoantibodies and recurrent miscarriage. Espinosa (20) studied on recent trends in the management of antiphospholipid syndrome. He believed despite the strong association between antiphospholipid antibodies and thrombosis and obstetric morbidity, their pathogenic role in the development of these clinical features has not been fully elucidated. Dr Adelowo et al. (21) explained antiphospholipid syndrome a thrombophilic condition, is being increasingly recognised as an important cause of recurrent pregnancy loss, preeclampsia and possible infertility. Dr. Velayuthaprabhu (12) and coworker analyzed the prevalence of anticardiolipin and antiphosphatidyl serine antibodies in relation to pregnancy failures in women with history of recurrent spontaneous abortion. They concluded anticardiolipin antibody is found to be the most important factor for recurrent abortion. In addition, women with negative anticardiolipin are having positive for another antiphospholipid antibodies, which may involve in recurrent abortion.

Mean anticardiolipin in case group was lower than control group but antipospholipid rate was equal in two groups. Antipospholipid antibodies based on number and gestational age of abortions were increased but anticardiolipin antibodies in all number of abortions almost were equal. Whatever the gestational age was higher in all number of abortion, mean antipospholipid antibodies was higher but anticardiolipine antibodies just was higher in relation with gestational age of first abortion. Almost mean antipospholipid and anticardiolipin antibodies in all patients were in high level just in first 5 years post all abortions but decreased after more than 5 years.
